# Funding and expenditure of a sample of community-based organizations in Kenya, Nigeria, and Zimbabwe

**DOI:** 10.1080/09540121.2013.764390

**Published:** 2013-06-09

**Authors:** Anya Krivelyova, Jakub Kakietek, Helen Connolly, Rene Bonnel, Brigitte Manteuffel, Rosalía Rodriguez-García, N'Della N'Jie, Andres Berruti, Simon Gregson, Ruchika Agrawal

**Affiliations:** a ICF International, Public Health, Atlanta, GA, USA; b World Bank, Washington, DC, USA; c Imperial College of London, London, UK

**Keywords:** HIV, AIDS, community-based organizations, funding, expenditure, Africa

## Abstract

Over the last decade, international donors, technical specialists, and governments have come to recognize the potential of community-based organizations (CBOs) in the fight against HIV/AIDS. Recent empirical studies suggest that community engagement, including the involvement of CBOs, adds value to the national response to HIV/AIDS. With the emerging evidence of the effectiveness of engaging communities in the fight against AIDS, it is crucial to understand the economic dimension of community engagement. This article provides an analysis of funding and expenditure data collected from CBOs in three African countries: Kenya, Nigeria, and Zimbabwe. It presents descriptive information regarding CBO funding and expenditure and examines the factors associated with the total amount of funds received and with the proportions of the funds allocated to programmatic activities and program management and administration. An average CBO in the sample received US$29,800 annually or about US$2480 per month. The highest percentage of CBO funding (37%) came from multilateral organizations. CBOs in the sample spent most of their funds (71%) on programmatic activities including provision of treatment, support, care, impact mitigation, and treatment services.

Over the last decade, international donors, technical specialists, and governments have come to recognize the potential of community-based organizations (CBOs) in the fight against HIV/AIDS. Recent pronouncements from key stakeholders in the global response to the epidemic emphasize the key role of community engagement (Schwartländer et al., 2011). The UNAIDS New Investment Framework for the Global HIV Response states that “community mobilization had been recognized as a cornerstone of HIV programmes” (UNAIDS, 2011, p. 6). The Community System Strengthening Framework adopted by the Global Fund to Fight AIDS, Tuberculosis, and Malaria (Global Fund) points out that “community support for health and social welfare has unique advantages in its close connection with communities, its ability to communicate through people's own culture and language and to articulate the needs to communities, and its ability to mobilize the many resources that community members can bring to the process of policy-making and decision-making and to service delivery” (Global Fund to Fight AIDS, Tuberculosis, and Malaria, 2010, p. iv).

Recent empirical evidence supports these pronouncements and suggests that community engagement, including the involvement of CBOs, adds value to the national response to HIV/AIDS. A review of evidence from 14 studies in eight countries in Africa and Asia found that community engagement was associated with increased mobilization of resources, improved knowledge, changes in sex behaviors, increased use of health services, and reduced HIV incidence and sexually transmitted infection (STI) prevalence in high-prevalence settings (Rodriguez-Garcia, Bonnel, & N'jie, 2012). An evaluation of the impact of CBO engagement in Kenya showed that community members in communities with stronger CBO engagement (operationalized as awareness of CBO activities in the community as reported in a household survey) knew more about HIV/AIDS and were more likely to report condom use than those in communities with weaker CBO engagement (Riehman et al., 2012). A similar evaluation in Nigeria found that community members in communities with stronger CBO engagement (operationalized as the number of CBOs per capita) reported higher availability and use of services related to HIV/AIDS (Kakietek et al., 2012).

With the emerging evidence of the effectiveness of engaging communities in the fight against AIDS, it is crucial to understand the economic dimension of community engagement. Assessing this dimension of CBO activity can improve our understanding of the amounts of domestic and international funding that reach local communities, of the types of sources from which CBOs receive their funding, of their vulnerabilities and resistance to fluctuations in the funding streams, and of the prospects for sustainability of the CBO response at the local level. A better understanding of the economic dimension of CBO engagement in the community response to HIV/AIDS is also necessary to assess whether the funds the CBOs receive are spent efficiently, what proportion of the funding is allocated to the activities that have the potential to benefit the community members, and what proportion is spent to benefit the CBOs themselves (e.g., staff salaries, other management costs).

Some research addressing funding and expenditure of civil society organizations (CSOs; but not necessarily specifically those that are community based and that engage local communities in the response to HIV/AIDS) have been conducted. However, it tends to be concerned with the flow of funds on the macro-scale (global or regional) and examines funding rather than expenditure (e.g., Foster, 2005; Kelly et al., 2006; Kelly & Birdsall, 2008; UNAIDS, 2009). Moreover, a significant portion of the available reports focuses narrowly on the funding provided by specific donors or groups of donors (e.g., World Bank, Global Fund, G8 countries; International HIV/AIDS Alliance, 2007; International HIV/AIDS Alliance and Global Fund, 2008; International HIV/AIDS Alliance, 2009; Kates, Boorts, Lief, Avila, & Gobet, 2010; Kates, Lief, & Avila, 2009). To date, no systematic assessment has been undertaken at the community level to examine sources from which CBOs receive financial support or how they allocate their funds. This article seeks to address this gap by providing an analysis of funding and expenditure data collected from CBOs in three African countries: Kenya, Nigeria, and Zimbabwe. It presents descriptive information regarding CBO funding and expenditure and examines organizational factors associated with the total amount of funds received and with the proportions of the funds allocated to programmatic activities and program management and administration. Comparing data from three countries collected using a uniform set of instruments allows us not only to asses inter-country differences but also, at the same time, to examine factors associated with funding and expenditure that hold across the three CBO country-specific samples (see below).

This study is part of a World Bank, Department of International Development (DFID), and UK Consortium on AIDS and International Development evaluation exercise to assess results achieved by community responses to HIV/AIDS.

## Methods

### Definitions

CBOs were defined as service organizations with involvement from community members that provide social services to local clients and included CSOs, nongovernmental organizations (NGOs), and faith-based organizations that target people infected with and affected by HIV and AIDS.

### Data collection

Information, regarding amounts and sources of funding and amounts and categories of expenditure, was collected from 126 CBOs: 26 in Kenya (in two provinces – Nyanza and Western), 67 in Nigeria (in six states: Adamawa, Akwa Ibom, Benue, Enugu, Kaduna, and Lagos, and the Federal Capital Territory[FCT]), and 33 in Zimbabwe (in two provinces – Manicaland and Matabeleland South) (Table 1; for details on community selection criteria, see Kakietek et al., 2012; Riehman et al., 2012). Data were collected through interviews with CBO staff and the review of available documents. In all three countries, a common funding and expenditure tool developed by ICF International was used for data collection.

**Table 1.** Number of interviewed CBOs and available data on funding, expenditures, and volunteers in the overall sample and in Kenya, Nigeria, and Zimbabwe.

Country	Regions	Number of CBOs interviewed	Complete funding data	Complete expenditure data
Full sample	N/A	126	116	109
Kenya	Two Provinces: Nyanza and Western Kenya	26	26	18
Nigeria	Six States: Adamawa, Akwa Ibom, Benue, Enugu, Kaduna, and Lagos and FCT	67	58	59
Zimbabwe	Two Provinces: Manicaland and Matabeleland South	33	32	32

### Sample selection

In Kenya and Nigeria, in each community, data were collected from up to six (Kenya) or eight (Nigeria) CBOs providing services related to HIV/AIDS. If the number of CBOs in a given community was less than or equal to six (in Kenya) or eight (in Nigeria), data were collected from all CBOs in that community. If the number of CBOs in the community was larger, the largest and the smallest CBO were included in the sample and additional CBOs were randomly selected from among the remaining CBOs providing HIV/AIDS-related services in that community. In Nigeria, on average, about 59% of the CBOs registered with the National and State Agencies for the Control of AIDS were interviewed in each community. In Kenya, on average, about 54% of the CBOs registered with the National AIDS Control Council and/ or local HIV/AIDS agencies in each community were interviewed.

In Zimbabwe, data were collected from organizations participating in the Manicaland M&E Facility Survey conducted as part of the Manicaland Project. Data were collected from all organizations that matched the operational definition of a CBO (see above). In Matabeleland, a list of organizations was provided by the District AIDS Coordinator. Data were collected from a convenience sample of organizations which matched our CBO definition.

### Data analysis

Reported amounts were annualized for all CBOs in the sample. All currencies reported were converted to US dollars. As data were collected in the three countries in different years (2009 in Kenya, 2010 in Nigeria, and 2011 in Zimbabwe), all US dollar amounts were deflated to 2011 values.

Data on volunteers and persons reached by the CBOs were collected using the same instrument as the funding and expenditure data. The interviewed CBOs staff reported how many volunteers their organization had and how many people it had reached in the past 12 months. Table 2 presents the summary statistics regarding the number of volunteers and persons reached in our sample of the CBOs.

**Table 2.** Summary statistics of the number of volunteers and persons reached by the CBOs in the past 12 months in the overall sample.

	Mean	SD	Median	Minimum	Maximum
Number of volunteers	85.98	196.69	21	1	1256
Number of persons reached in the past 12 months	9685.76	38,985.3	575	12	293,072

Regression models were used to examine the association between organizational factor for which data were collected as part of the study and the amount of funding and expenditure. To correct for heteroscedasticity and autocorrelation resulting from the CBOs clustered within the same countries and communities, we estimated regression models with robust standard errors (Fox, 1997). In the model where the total amount of funding was the outcome variable, explanatory variables included the country in which the CBOs were located, the sources from which they received the funding, and number of volunteers they had. In the models where the percentage of expenditure was the outcome variable, explanatory variables included the country in which the CBOs were located, total funding CBOs received, sources of funding, and number of volunteers and number of persons reached within the past 12 months.

All analyses were conducted using STATA 11 (STATA Corp., College Station, TX, USA).

Data on volunteers were available for 84 CBOs and an average CBO had 86 volunteers. Data on persons reached were available for 84 CBOs and average CBO had reached 9686 individuals. Note that the arithmetic means reported here are inflated due to the presence of outliers.

## Findings

### CBO funding

An average CBO in the sample received US$29,800 annually (standard deviation (SD): US$53,471). An average CBO in Zimbabwe received about US$72,000 (SD: US$75,114), compared to US$10,000 (SD: US$7783) in Kenya and US$16,000 in Nigeria (SD: US$37,269). The difference may be due to differences in the types of organizations included in the sample in Zimbabwe and the other two countries (see Discussion section). High SDs of the mean levels of funding, both in the overall sample and in the individual countries, demonstrate substantial variation in the funding reported by individual CBOs. Median levels of funding, which are less susceptible to the influence of outliers, were: US$ 9732 (Inter-quartile range [IQR]: US$ 2294–US$30,000) in the overall sample; US$ 9486 (IQR: US$4498–US$13,034) in Kenya; US$2851 (IQR: US$1540–US$12,103) in Nigeria; and US$ 53,847 (IQR: US$24,936–US$96,013) in Zimbabwe.

**Figure 1.**
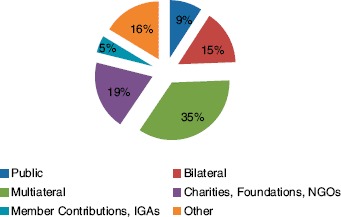
Percentage of total funding received from specified sources of support.

The funds came from public sources (national AIDS coordinating bodies, Ministries of Health; other line ministries; state, provincial, and local authorities); bilateral sources (e.g., USAID, DFID); multilateral sources (e.g., Global Fund, World Bank, UNAIDS); charities, foundations, and larger NGOs; and member contributions and income-generating activities (IGAs) (Figure 1).

**Figure 2.**
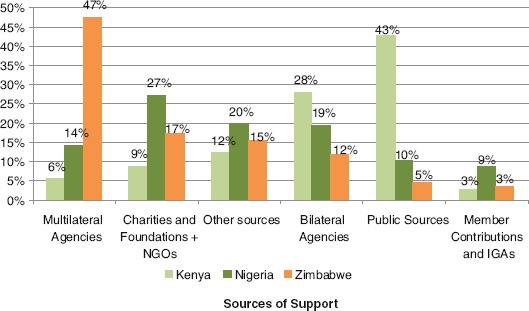
Percentage of total funding received from specified sources of support in Kenya, Nigeria, and Zimbabwe.

About 69% of total funds reported by CBOs came from international sources. The highest percentage of funding for CBOs in the sample came from multilateral organizations (35%). Only 5% came from member contributions and IGAs, even though 29.7% of CBOs reported receiving financial support from that source. Distribution of funding sources in each of the three countries is presented in Figure 2.

There was little diversification in the types of funding sources. More than half of the CBOs (50.9%) reported receiving support from only one type of funding source (Table 3).

**Table 3.** Number and percentage of CBOs receiving support from different number of funding source types.

	CBOs receiving support from a specific number of source types	
Number of different types of sources	*N*	%
1	60	50.90
2	39	33.10
3	16	13.60
4	3	2.50

Regression analysis showed that CBOs funded by multilateral organizations received on average about US$27,535 more annually than organizations that did not receive funding from that source. Receiving funding from other types of sources (i.e., bilateral, public, member contributions/IGAs, and others) was not associated with the amount of funding received. In addition, sample CBOs located in Zimbabwe received significantly more funds that those located in Nigeria (reference group) and Kenya. The difference in funding between Nigerian and Kenyan CBOs included in the sample was not statistically significant (see Table 4).

**Table 4.** Results of a multiple regression model of predictors of per CBO annualized funding.

	Total annualized funding per CBO β (SE)
CBO located in^a^	
*Kenya*	− 1882.81 (9797.50)
*Zimbabwe*	39,607.87* (10,584,05)
CBO received funding from	
*Public sources*	12,615.37 (10,034.11)
*Bilateral sources*	10108.80 (8920.67)
*Multilateral sources*	27,534.87* (8274.92)
*Charities, foundations, and NGOs*	22,189.85* (8183.88)
*Member contributions and IGAs*	1206.67 (8139.26)
*Other sources*	2562.58 (8331.45)
Number of volunteers	25.32 (26.35)
Constant	− 7942.07 (9891.6)
*N*	105
Adjusted *R*^2^	0.296
*P*(F)	<0.001

Notes: ^a^CBOs located in Nigeria are the reference category.

* *p* < 0.05.

### CBO expenditure

**Figure 3.**
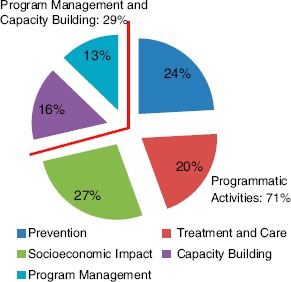
CBO expenditure.

CBOs in the evaluation sample spent about 71% of their funds on programmatic activities aimed at providing services to local populations affected by and infected with HIV (Figure 3). Within that broad category, the highest proportion of total expenditure of funds was spent on support and mitigation of the economic impact of AIDS (27%), followed by prevention (24%) and treatment and care (20%). The remaining 29% of funds were spent on capacity building and program management and administration (16% and 13%, respectively). A list of specific expenditure sub-categories included in each of the categories listed above is available in the Appendix 1.

**Figure 4.**
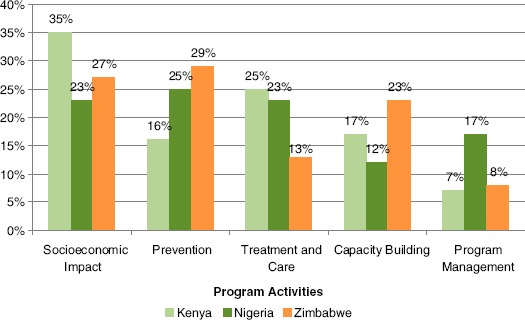
Percentage of CBO expenditures allocated to each expenditure category in Kenya, Nigeria, and Zimbabwe.

Funding allocation in each of the three countries in presented in Figure 4.

Regression analysis showed that the percentage of funds spent on program management was significantly associated with the CBO's total funding received; total number of volunteers; total number of clients reached; and receipt of funding from charities, foundations and NGOs and member contributions/IGAs) (Table 5).^1^ Each additional volunteer was associated with 0.04 percentage point of additional spending on program management. Every additional 100 clients served in a given year was associated with a 0.02 percentage point increase in spending on program management. CBOs that received funding from member contributions and IGAs spent about 14.1% less on program management than CBOs that did not receive funding from that source. Each additional US$1000 of total funding a CBO received regardless of the sources, was associated with a decrease of about 0.5 percentage point in the percentage of funds allocated to program management. Finally, CBOs that reported receiving funding from charities, foundations, and NGOs spent on average 18.7% more on program management than CBOs that did not receive funding from that source. Receiving funding from other types of funding sources was not associated with the percentage of funds spent on program management. The differences among the three countries were not statistically significant (Table 5).

**Table 5.** Results of multiple regression models of predictors of CBO expenditures on program management and programmatic activities.

	Program management^a^ β (SE)	Programmatic activities β (SE)
Country^b^		
*Nigeria*	0.1305 (0.10)	− 0.0654 (0.09)
*Kenya*	0.0858 (0.11)	− 0.0819 (0.16)
Total funds received	− 4.4700E-06** (2.02E-06)	− 1.44E-06 (3.05E-06)
CBO funded by		
*Public sources*	− 0.0978 (0.10)	− 0.0338 (0.09)
*Bilateral sources*	− 0.0863 (0.07)	−0.0526 (0.09)
*Multilateral sources*	− 0.0725 (0.06)	−0.0277 (0.07)
*Charities*, foundations, and NGOs	0.1853** (0.09)	0.0276 (0.09)
*Member contributions and IGAs*	− 0.1433** (0.07)	−0.0117 (0.07)
*Other sources*	− 0.0865 (0.07)	−0.0361 (0.08)
Number of clients reached	2.06E-06* (1.13E-06)	1.80E-06 (1.68E-06)
Number of volunteers	3.9100E-04** (1.38E-04)	− 1.08E-04 (2.21E-04)
Constant	0.2873** (0.10)	0.7944*** (0.11)
*N*	50	58
*R*^2^	0.42	0.12
*P*(F)	<0.001	0.3

Notes: ^a^Only CBOs that reported expenditures on program management and administration were included in the models where program management was dependent variables. It was assumed that zero expenditure in this area was due to errors in reporting.

b CBOs located in Zimbabwe are the reference category.

* *p* < 0.1; **p < 0.05; ***p < 0.01.

None of the factors included in the model were associated with the percentage of spending allocated to programmatic activities (prevention, treatment and care, mitigation of the socioeconomic impact of AIDS).

## Discussion

An average CBO in the sample received US$29,800 annually. The data showed a significant difference in the level of annualized funding between CBOs in Zimbabwe (US$72,100) and Nigeria and Kenya (US$16,300 and US$10,300, respectively). It is possible that Zimbabwean CBOs included in the sample were larger and better able to mobilize resources. However, regression model that examined the factors associated with the amount of funding showed no significant association between CBO size, measured as the number of volunteers, and the amount of funding received. Therefore, other organizational factors, such as prevailing types of CBO structures (e.g., grass-root informal organizations versus highly formalized hierarchical organizations) and contextual factors (legal and political context within which CBOs operate, linkages and relationships between COBs and governmental and donor agencies) not captured by the data collected, are behind the difference in the CBO funding between Zimbabwe and the other two countries.

The lowest percentage of total CBO funding (5%) came from member contributions and IGAs. Our findings corroborate the extant literature, which shows that CSOs providing services related to HIV/ AIDS rely primarily on international support (e.g., Bonnel et al., 2011; Unom and Monye, 2010). This is not surprising given that CBOs in our sample work in countries and communities with high levels of poverty and severe resource constraints. However, our findings also suggest that engaging community members in CBOs’ work has benefits that go beyond increased funding. We found that CBOs that received funds from member contributions and IGAs devoted less of their funding to administration and program management than CBOs that did not receive support from within their communities. Although this study cannot establish any causal linkages, it is possible that community members who have a financial stake in the CBOs working in their community have more incentives or are better able to monitor CBO activities, keep CBOs accountable, and ensure that the funding is allocated efficiently and serves to provide services to the community. Normative arguments have been made that community engagement in CBO work is beneficial because it increases CBO legitimacy and community buy-in (UNAIDS, 2011; Global Fund, 2010). Our findings add an economic dimension to those arguments and suggest that community engagement can help ensure that CBOs direct smaller proportions of their funding to overhead.

We found little diversification in the types of sources from which CBOs receive financial support. It is possible that CBOs tend to specialize and target only specific types of funding agencies. It is also possible that funding from some sources is available only to some CBOs in some communities. Regardless of its causes, the lack of diversification can make CBOs vulnerable to fluctuations in international funding which can be very significant, especially given the recent global economic climate. For example, in 2007, Zimbabwe received US$62.7 million from bilateral sources to support its response to HIV/AIDS. In 2008, this funding dropped by more than 90% to US$5.5 million (Government of Zimbabwe, 2009).

The highest percentage of CBO funding (37%) came from multilateral organizations. However, only one-third of CBOs received support from that source. Our findings showed that those that did reported about US$27,500 more in annual funding than CBOs that did not receive support from that source. It is possible that multilateral organizations distribute their funding selectively to specific types of CBOs (e.g., larger or better known CBOs). Another possibility is that while multilateral funding is available to all CBOs, only some chose to or can tap into that source of funding. Further inquiry is needed to determine why some CBOs receive funding from multilateral donors while others do not.

CBOs in the sample spent most of their funds (71%) on programmatic activities. This is encouraging and suggests the bulk of CBO funding ultimately reaches community members in the form of services provided. However, program management and administration expenditures accounted for 13% of total spending – substantially more than those reported in the National AIDS Spending Assessments for Kenya and Zimbabwe (Government of Zimbabwe, 2009; Government of Kenya, 2009). Those findings contrast with arguments found in the literature on CSOs and human development, which posit that CSOs are more efficient and have lower overhead costs than governmental bureaucracies (e.g., Clark, 1995).

We found that CBOs receiving support from foundations, charities, and larger NGOs tended to spent more on program management than CBOs that did not receive funding from these sources. It is possible that funding from these sources comes with fewer restrictions on how funds can be spent, and CBOs can direct more of this funding to support program management. It is also possible that this type of funding requires higher administrative costs. This is particularly likely if one CBO receives funding from several charities, foundations, and NGOs and is required to submit different reports with different information and indicators to different funding organizations.

This study did not assess whether and to what extent CBO expenditure priorities matched the true community needs. Limited discussion of the way in which a sample of CBO interviewed in Nigeria selected the activities in which they engaged and whether those activities corresponded with the epidemic profiles of the communities can be found in Kakietek et al. (2012). An examination of the match between CBO engagement and community needs must be a key element of future research on the contributions of CBOs to the community response to HIV/AIDS.

It is possible that the factors affecting funding and expenditure were different in Kenya, Nigeria, and Zimbabwe. Unfortunately, the small sample size (especially for Kenya and Zimbabwe, where the number of observations and the degrees of freedom were less than 30) did not allow us to estimate three separate country-specific regression models to assess whether and to what extent different factors influenced funding and expenditure in each country.

Information on funding and expenditure was provided by the CBOs on a voluntary basis. We do not believe that a formal audit of the CBO accounts to assess the validity of the reported data was possible. We did assess the consistency of the information provided (whether the amounts and percentages reported for specific expenditure and funding categories add up to the reported totals) as part of the data cleaning and entry process and followed up with the CBOs to clarify any discrepancies we found.

One limitation of this study is that it uses a convenience sample of CBOs and therefore it cannot be considered representative of all CBOs in Zimbabwe, Nigeria, and Kenya. To our knowledge, no systematic data exist on the number of CBOs engaging in HIV/AIDS-related activities in any of the three countries. Therefore, it is impossible to estimate the proportion of the CBOs in each country that was included in our sample. The total number of CBOs and the percentages of CBOs interviewed (Table 1) were compiled during stakeholder meetings convened as part of this project and were limited to the states/provinces and communities included in the evaluation.

Another limitation is that our analysis does not capture potentially important contextual and organizational factors that may affect how much funds CBOs receive and how they spend them (this may be reflected in the relatively low adjusted *R*^2^ values of the regression models). However, our intention was to examine the associations between funding, expenditure, and other selected characteristics of the CBOs (e.g., size and reach) rather than to build a comprehensive predictive model of CBO funding and expenditure patterns.

Finally, our sample covers a limited geographic area in each of the three countries. Nevertheless, to our knowledge, this is the most comprehensive crosscountry data-set on CBO funding and expenditure collected at the community level using a consistent methodology. As such, it provides a stepping stone for a more systematic assessment of sources from which CBOs receive financial resources and how they allocate them.

## Note

1. As mentioned above, the distribution of the total funding in the sample was skewed. As robustness checks, we re-estimated the model using the square root and the natural log of the total amount of funding as dependent variables to make the distribution of the dependent variable in the model better approximate the normal distribution. The results of the model with the square root of total funding were virtually identical with the results of the model with untransformed funding levels with the same covariates significant at the same significance levels. In the model where the natural log of the total amount of funding was used as the dependent variable, the p value of the multilateral sources of funding indicator was slightly higher (0.053 compared to 0.019), but the remaining independent variables significant in the model with the untransformed total funding were significant at the same significance level in the natural logarithm model. Also, in that model, the difference in funding between CBOs in Kenya and Nigeria was statistically significant.

## Appendix 1: Expenditure sub-categories

**Table:**

Expense category	Subcategories included
HIV preventive service provided	• Voluntary counseling and testing (VCT) • AIDS education in schools and HIV information through resource centers • Screening blood for HIV infection • Male circumcision • Communication for social and behavior change • Social marketing of condoms (education, distribution, and use) • Development and distribution of information, education, and communication materials • Use of mass media • Commercial sex worker peer education • Prevention measures among injecting drug users • Prevention, diagnosis, and treatment of sexually transmitted infections (STIs) • Prevention of HIV transmission aimed at persons living with HIV/AIDS (PLWHA) • Programs for men who have sex with men • Prevention of mother-to-child transmission (PMTCT) • Other HIV preventive services
HIV treatment and care service provided	• Clinical care • Counseling • Lab tests (e.g., CD4, viral loads) • Anti-retroviral therapy (ART); herbal therapy and nutrition • Other drugs (e.g., opportunistic infection, painkillers) • Home-based care • Other HIV treatment and care services
Socioeconomic impact	• OVC support • Physiological support for grandmothers • Income generating activities (IGA) programs • Group savings and loan programs
Capacity building	• Training of community groups and staff • Community monitoring and evaluation (M&E) activities • Ministry of health support and networking collaboration • Complementary programs (e.g., agriculture groups) • Monetary incentive for human resources • Other capacity building programs
Program management and administration	• Planning, coordination, and administration • Monitoring and evaluation • Supply chain system • Other administration services
